# Ephexin3/ARHGEF5 Together with Cell Migration Signaling Partners within the Tumor Microenvironment Define Prognostic Transcriptional Signatures in Multiple Cancer Types

**DOI:** 10.3390/ijms242216427

**Published:** 2023-11-17

**Authors:** Dante Gustavo Juan-Guadarrama, Yarely Mabell Beltrán-Navarro, Guadalupe Reyes-Cruz, José Vázquez-Prado

**Affiliations:** 1 Department of Pharmacology, Cinvestav-IPN, Av. Instituto Politécnico Nacional 2508, Col. San Pedro Zacatenco, Mexico City 07360, Mexico; 2 Department of Cell Biology, Cinvestav-IPN, Av. Instituto Politécnico Nacional 2508, Col. San Pedro Zacatenco, Mexico City 07360, Mexico

**Keywords:** Ephexin3, Rho guanine nucleotide exchange factor (RhoGEF), cancer signaling signature, cancer datasets mining, TCGA, DepMap, synthetic lethality datasets

## Abstract

Cancer cell migration involves a repertoire of signaling proteins that lead cytoskeleton reorganization as a critical step in metastatic dissemination. RhoGEFs are multidomain effectors that integrate signaling inputs to activate the molecular switches that orchestrate actin cytoskeleton reorganization. Ephexins, a group of five RhoGEFs, play oncogenic roles in invasive and metastatic cancer, leading to a mechanistic hypothesis about their function as signaling nodes assembling functional complexes that guide cancer cell migration. To identify clinically significant Ephexin signaling partners, we applied three systematic data mining strategies, based on the screening of essential Ephexins in multiple cancer cell lines and the identification of coexpressed signaling partners in the TCGA cancer patient datasets. Based on the domain architecture of encoded proteins and gene ontology criteria, we selected Ephexin signaling partners with a role in cytoskeletal reorganization and cell migration. We focused on Ephexin3/ARHGEF5, identified as an essential gene in multiple cancer cell types. Based on significant coexpression data and coessentiality, the signaling repertoire that accompanies Ephexin3 corresponded to three groups: pan-cancer, cancer-specific and coessential. To further select the Ephexin3 signaling partners likely to be relevant in clinical settings, we first identified those whose high expression was statistical linked to shorter patient survival. The resulting Ephexin3 transcriptional signatures represent significant accumulated risk, predictive of shorter survival, in 17 cancer types, including PAAD, LUAD, LGG, OSC, AML, KIRC, THYM, BLCA, LIHC and UCEC. The signaling landscape that accompanies Ephexin3 in various cancer types included the tyrosine kinase receptor *MET* and the tyrosine phosphatase receptor *PTPRF*, the serine/threonine kinases *MARK2* and *PAK6*, the Rho GTPases *RHOD*, *RHOF* and *RAC1*, and the cytoskeletal regulator *DIAHP1*. Our findings set the basis to further explore the role of Ephexin3/ARHGEF5 as an essential effector and signaling hub in cancer cell migration.

## 1. Introduction

Ephexins are a subgroup of five DH-RhoGEFs implicated as oncogenic effectors in various cancer types [1,2]. The group was named after Ephexin1, cloned as a neuronal interactor of EphA4 receptors, necessary to regulate axonal cone dynamics [3]. Most Ephexins are documented partners of EphA receptors controlling cell interactions and guidance processes elicited by Ephrins, their coreceptors in neighboring cells [4]. By activating different Rho GTPases, all Ephexins are involved in signaling cascades leading cytoskeletal reorganization and cell migration [1]. Ephexins 1, 2 and 3, also known as NGEF, ARHGEF19, and ARHGEF5, respectively, preferentially activate RhoA [3,5,6], while Ephexin4, also named ARHGEF16, activates RhoG [7], and Ephexin5, encoded by *ARHGEF15*, activates Cdc42 [8]. In various cancer types, Ephexins have been detected as highly expressed transcripts, and abundant or highly phosphorylated proteins that are correlated, by themselves or with coexpressed signaling partners, with shorter patient survival. Examples include Ephexin1 in lung and colorectal cancers [9,10], Ephexin2/ARHGEF19 combined with markers of epithelial–mesenchymal transition in lung cancer [11], Ephexin3/ARHGEF5 combined with Src, in non-small cell lung cancer [12], and Ephexin5/ARHGEF15 in pancreatic cancer [13]. 

Preclinical experimental evidence has mechanistically linked Ephexins to tumor growth and metastasis, particularly studying cancer cell lines in which Ephexins have been knocked down with the aim to assess whether or not their expression is required for tumor growth in mice [1]. In protumoral bone marrow-derived cells, Ephexin3/ARHGEF5 is highly expressed [14]. At the molecular level, various signaling proteins with paramount importance in cancer progression, such as Ras, Raf, Akt, Src, and growth factor receptors, use Ephexins as effectors in cell invasion, migration, and mesenchymal transition, among other processes linked to cancer progression [2,7,10,11,15]. As oncogenic effectors, these sophisticated RhoGEFs, characterized by a phylogenetically conserved catalytic DH-PH module, which in four of them is followed by a carboxyl terminal SH3 domain, and preceded by a variable amino terminal regulatory region [1], occupy strategic positions assembling protumoral signaling complexes. Accumulating evidence indicates a potential clinical impact on cancer progression of Ephexins and their signaling partners. For instance, in lung and colorectal cancer cells, Ephexin1 works as an Akt-dependent Ras effector [10], and serves as a bridge between EGFR and EphA to mediate oncogenic effects driven by EGFR [9]. In mouse models of lung cancer, Ephexin2 expression is required for tumor growth. The implicated signaling complex involves BRAF and HRAS, activating the Erk pathway [11,16]. A functional cloning strategy looking for active oncogenes led to the identification of a truncated version of Ephexin3 (ARHGEF5/TIM) as a transforming oncogene [17]. Further studies revealed that Ephexin3 is linked to the invasive capacity of cancer cells forming a signaling complex with Src kinase, PI3K, and cortactin, a filamentous actin-binding protein, driving the assembly of invasive structures [18]. Furthermore, Ephexin3 interacts with the signaling adaptors Shc1 [19], and GRB2 [20], is phosphorylated by EGFR [21], and contributes to the mesenchymal–epithelial transition caused by TGFβ [15]. Ephexin4 promotes breast cancer cell migration as part of a signaling complex with EphA2, EGFR, ELMO, DOCK1, RhoG, and Rac1 [7], and promotes cell survival as an effector of EphA2 and Akt [22]. Ephexin4 interacts with Fyn, a tyrosine kinase of the Src family, promoting cell survival, proliferation, migration and invasion [23]. In glioma cells, the GLI2 oncogene promotes cell migration by enhancing Ephexin4 expression [24]. Ephexin5 regulates angiogenesis by activating Cdc42 [8]. 

The molecular context in which oncogenic signaling by Ephexins plays a role in cancer progression is putatively different in distinct cancer types and tumor microenvironments. As a rational strategy to find clinically relevant Ephexin signaling partners among the enormous amounts of biological information available in cancer omic datasets, we focused on signaling proteins with a role in cell migration, based on the hypothetical role of RhoGEFs such as Ephexins as signaling platforms integrating migratory cues linked to cancer dissemination. Since we identified Ephexin3 as essential in multiple cancer cell lines, we focused on the identification of its signaling companions, relevant in the context of cytoskeletal dynamics and cell migration, together with the analysis of cancer cell co-vulnerabilities, and screened the signaling repertoire linked to unfavorable prognosis according to the statistical analysis of the mRNA expression and overall survival of patients with different types of cancer. 

## 2. Results

### 2.1. Expression of Ephexins in Various Cancer Types 

We analyzed The Cancer Genome Atlas (TCGA) datasets (https://www.cancer.gov/tcga, accessed on 1 November 2021), which include information of about 11,000 patients corresponding to 32 types of cancer. Publicly available information includes exome sequencing, gene copy variations, transcriptomics, proteomics, and phosphoproteomics, together with the respective clinical outcomes [25,26,27,28]. Furthermore, aiming to identify cancer cell vulnerabilities involving Ephexins, we integrated our analysis with synthetic lethality datasets from The Cancer Dependency Map, or DepMap (https://depmap.org/portal/, accessed on 1 November 2021) that are based on the systematic use of RNAi, CRISPR-Cas9 and pharmacological inhibitors applied to hundreds of cancer cell lines [29,30,31]. 

**Figure 1:**
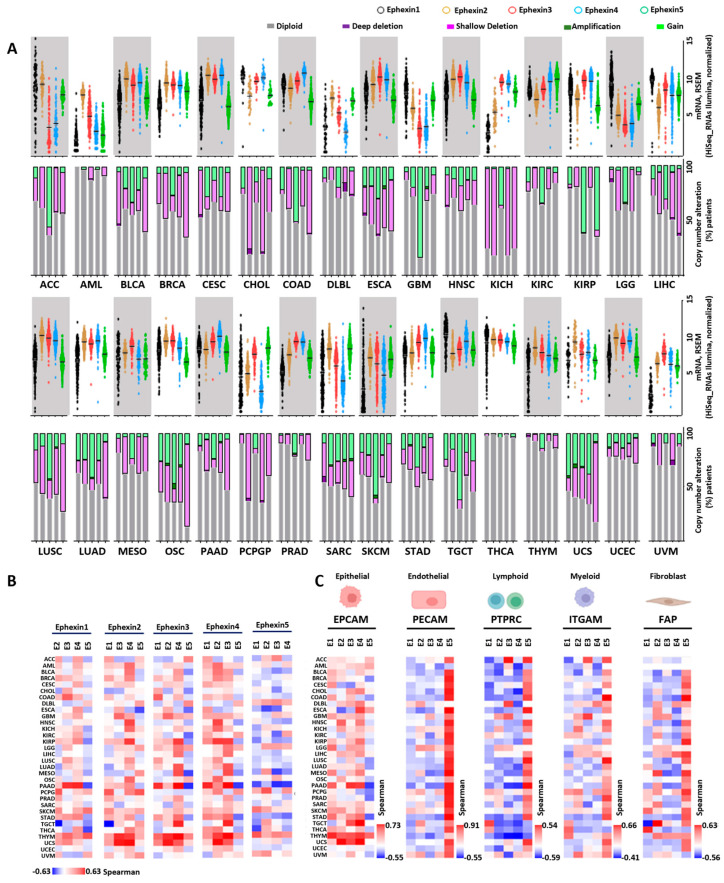
Expression of Ephexins in 32 cancer types and their coexpression patterns. (**A**) Expression of Ephexins (1–5) shown in scatter plots per type of cancer. Percentage of copy number alterations of each Ephexin in bar graphs per type of cancer. Color codes for Ephexins and types of alterations in the upper region. (**B**) Correlation between Ephexins in the 32 types of cancer that are part of the TCGA project. (**C**) Correlation of Ephexins (1–5) with stromal cell markers. *EPCAM*, *PECAM*, *PTPRC*, *ITGAM* and *FAP* for epithelial, endothelial, lymphoid, myeloid and fibroblast cell type markers, respectively.

In order to compare the transcriptomic expression and genomic characteristics of Ephexins in multiple human cancer types, we used the cBioportal platform [32] to analyze 32 TCGA datasets including 10,967 patients. Figure 1A shows that all five Ephexins were detected in the 32 cancer types. Although some patients had copy alterations in the Ephexin genes, as shown in the bar graphs, no clear correlation between gene copy number alteration and mRNA expression was apparent. 

### 2.2. Coexpression Patterns of Ephexins among Themselves and with Markers of Tumor Stromal Cells

Given their structural similarity, if they are coexpressed, Ephexins might have redundant functions. We thus investigated their potential coexpression by looking into the Spearman correlation coefficients among Ephexins in the 32 cancer types. As shown in Figure 1B, Ephexins 1, 2, 3 and 4 were positively coexpressed in most cancers, as indicated by the prevalence of red areas marking positive correlations in the heatmap, while Ephexin5 presented a pattern of negative correlation with other Ephexins (Figure 1B, blue areas in the fifth heatmap). Since all Ephexins were expressed in the 32 cancer types and presented various patterns of correlation among them, we investigated how their expression correlated with markers of the tumor stroma. As shown in Figure 1C, in the first heatmap, Ephexins 1, 2, 3 and 4 positively correlated with *EPCAM*, an epithelial cell marker, and exhibited a negative correlation with markers of various cells of the tumor microenvironment, such as endothelial, lymphoid, myeloid and fibroblasts, as indicated by the blue areas corresponding to a negative correlation with *PECAM*, *PTPRC*, *ITGAM* and *FAB*, respectively (Figure 1C, heatmaps 2–5). On the other hand, Ephexin5 correlated with different markers of stromal cells, including endothelial, lymphoid, and myeloid cells, and fibroblasts (Figure 1C, heatmaps 2–5), but negatively correlated with *EPCAM* (Figure 1C, heatmap 1).

### 2.3. Cancer Cell Lines Sensitive to Ephexin3 Knock-Out

**Figure 2:**
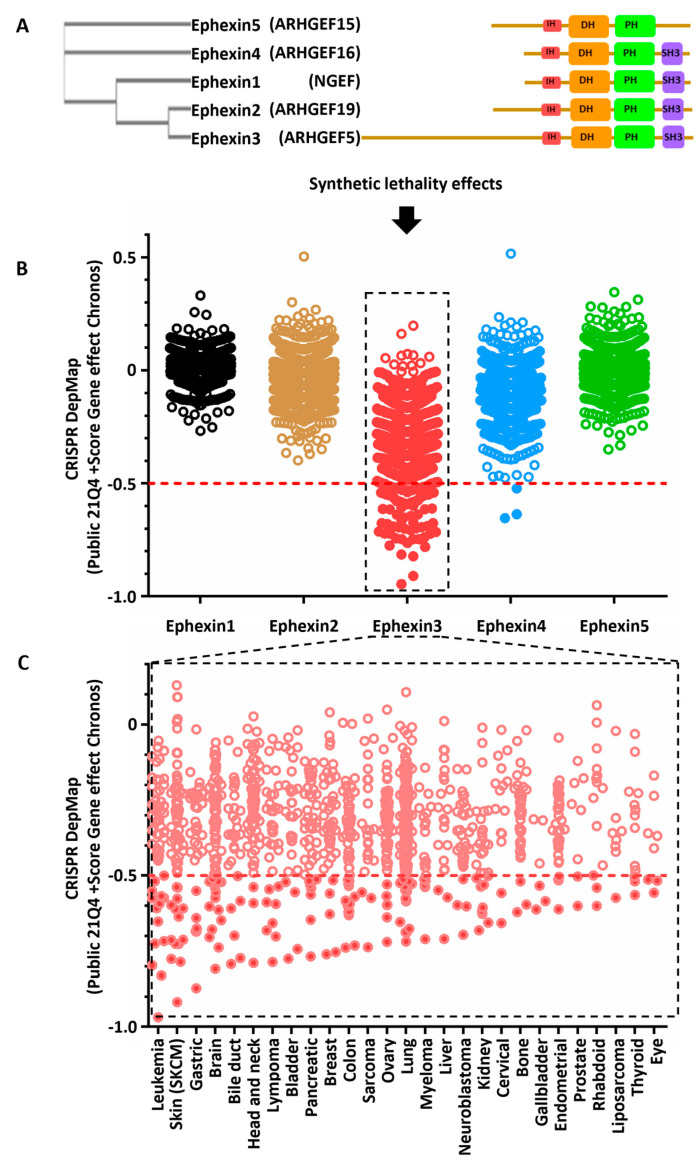
Cancer cell lines sensitive to Ephexin3 knock-out. (**A**) Phylogenetic tree based on the homology between the Ephexins’ catalytic domain and representations of their structures. (**B**) Ephexin gene dependency of cancer cell lines by loss of function (CRISPR). T statistic cut off value less than −0.5. (**C**) Tissues of origin of the cell lines that were sensitive to the loss of function of the Ephexin3 gene. Ephexin3-dependent cell lines are indicated by filled circles.

To address whether or not Ephexins, expressed in multiple cancer types, were involved in oncogenic signaling cascades necessary for cell viability, we analyzed synthetic lethality datasets, using the DepMap platform (https://depmap.org/, accessed on 1 November 2021) [29,31], looking for cancer cell lines in which Ephexins were essential. In the event of redundancy in their functions, phylogenetically closest Ephexins might putatively be functionally redundant. Ephexins 1, 2 and 3 are the most closely related members of the family, while Ephexins 4 and 5 were the least conserved, as indicated by the phylogenetic tree obtained from a multiple alignment of the catalytic region (Figure 2A). Ephexin3 was particularly relevant in cancer cell lines of different cancer types, with a T statistic value less than or equal to −0.5 (Figure 2B). Given the differential essentiality properties of Ephexin3, we integrated these data with the information that we obtained from the TCGA transcriptomic datasets. Ephexin3 essentially was found in cell lines corresponding to 27 different tissues (Figure 2C). Importantly, not all cell lines from a particular origin were vulnerable to the lack of Ephexin3, indicating the potential existence of specific oncogenic landscapes linked to Ephexin3 essential signaling.

### 2.4. Ephexin3 Signaling Partners Linked to Cell Migration and Cytoskeleton Reorganization in 32 Cancer Types

**Figure 3:**
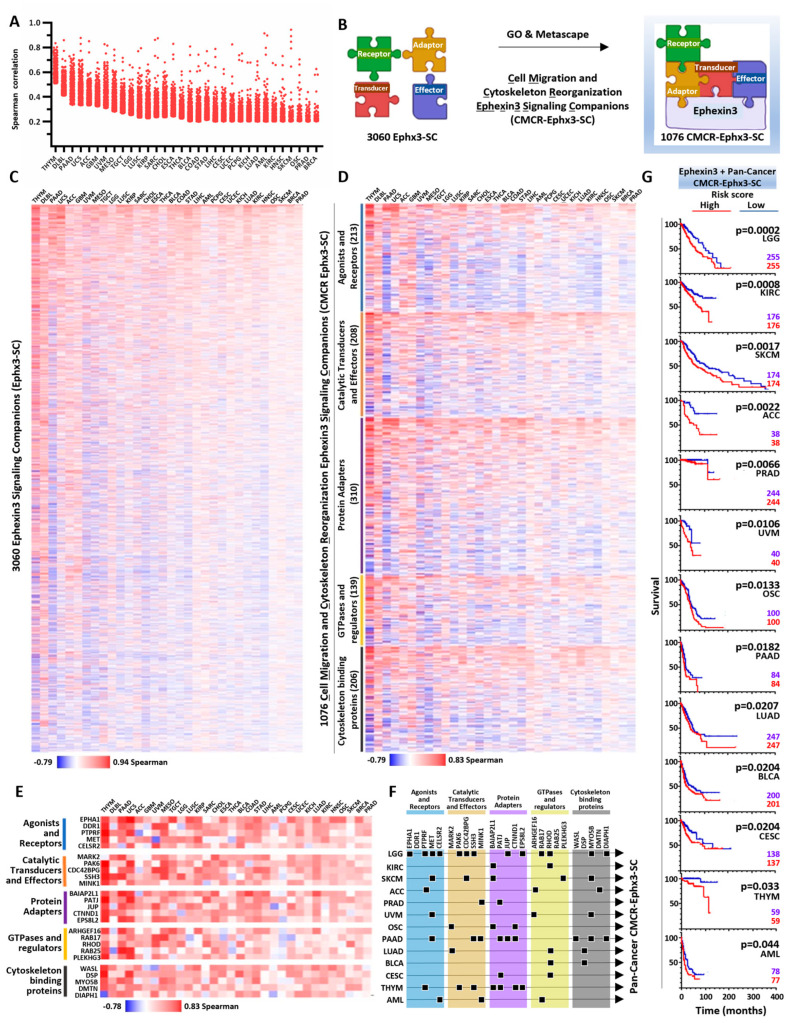
PanCancer Ephexin3 signaling partners linked to cell migration and cytoskeleton reorganization (CMCR-Ephx3-SC); transcriptional signatures are linked to shorter patient survival. (**A**) Ephx3-SC repertoire and their Spearman’s correlation values in the 32 types of cancer. Spearman’s coefficients of the top 10% and/or of at least 0.2. (**B**) Model showing the screening of Ephx3-SCs through Gene Ontology and Metascape to obtain genes related to cell migration and cytoskeleton reorganization. (**C**) Heatmap illustrating 3060 Ephx3-SCs in the 32 cancer types. Columns ordered according to the sum of positive values. (**D**) Heatmap showing 1076 CMCR-Ephx3-SCs in the 32 cancer types organized according to structural criteria. The upper rows for each category are the most correlated. (**E**) Top five correlated genes per signaling category in the 32 cancer types. (**F**) Binary map showing the signaling companions’ individual correlation with shorter survival in different types of cancer. Color blocks define the signaling categories. Arrows join the genes included in signaling signatures of the indicated cancer studies. (**G**) Survival curves of PanCancer transcriptional signatures with a high-risk score in 13 cancer types. Graphs ordered according to statistical significance. Patients were segregated according to median expression. Multivariate Cox regression analyses were conducted in KM plotter.

Ephexin3 essentiality in various cancer cell lines indicated its potential role in oncogenic cascades. We then focused on the identification of Ephexin3 signaling companions in the TCGA transcriptomic datasets. For each cancer type, we obtained about 19,500 Spearman correlation values of transcripts indicating negative, null, or positive coexpression with Ephexin3. We tagged the transcripts that encode cell signaling proteins, resulting in a list of about 4000 for each cancer type. We then selected those with the highest positive values, within the top 10% Spearman values of correlation. In the cases in which the top 10% contained transcripts with Spearman correlation values below 0.2, we used this value as the cutoff. With this strategy, we identified about 330 Ephexin3 signaling companions per cancer type (Figure 3A). We generically called them Ephx3-SC. Since most transcripts with a higher correlation with Ephexin3 differed among the different cancer types, the list joining all the signaling companions of the 32 cancer types included 3060 different Ephx3-SCs. To select those coding for proteins involved in cell migration and cytoskeleton reorganization (CMCR), we used Gene Ontology criteria and evolutionary relationships among protein families. We analyzed the data using the Panther platform (https://pantherdb.org/, accessed on 26 July 2022) [33], followed by manual curation. As a confirmatory strategy, the curated list was analyzed on the Metascape platform (https://metascape.org/, accessed on 11 October 2022) [34]. The most enriched GO tags found in Metascape included those that define processes linked to cell migration and the reorganization of the cytoskeleton such as chemotaxis, regulation of cell migration, the organization of the cytoskeleton and the regulation of processes based on F-Actin. With this additional criterion, we selected 1076 transcripts (calling them CMCR-Ephx3-SC) coding for receptors, transducers and effectors, among others (Figure 3B). The initial 3060 Ephx3-SCs were represented in a heatmap where the Spearman correlation values of all of them, either positive or negative, in all the 32 cancer types, were organized according to the sum of positive values (Figure 3C). The Ephexin3 signaling companions linked to cell migration and cytoskeleton reorganization (CMCR-Ephx3-SC) were organized based on five functional signaling properties: (1) agonist and receptors, (2) catalytic transducers and effectors, (3) protein adapters, (4) small GTPases and their regulators, and (5) cytoskeleton binding proteins; these are represented in the heatmap shown in Figure 3D. It was clear that some of these signaling partners, found at the top of each category, corresponded to components of the signaling landscape that joins Ephexin3 in various cancer types, which might integrate relevant pan-cancer pathways involved in cell migration and cytoskeleton reorganization (Figure 3D). 

### 2.5. PanCancer CMCR-Ephx3-SC Defining Transcriptional Signatures Statistically Linked to Shorter Patient Survival

Of the 1076 CMCR-Ephx3-SCs, we selected the top five of each signaling group (Figure 3E). Using the KM plotter platform (https://kmplot.com/analysis/, accessed on 30 November 2022) [35], we analyzed the Kaplan–Meier survival curves of these 25 PanCancer CMCR-Ephx3-SCs in the 32 cancer types. Those whose higher expression significantly correlated with shorter patient survival, indicated with squares in Figure 3F, became part of the transcriptional signatures for the cancer types indicated as arrows joining the squares in Figure 3F. All other cancers not indicated here did not have PanCancer CMCR-Ephx3-SCs within the top five of each signaling group that correlated with patient survival. We evaluated whether or not these transcriptional signatures, which also included Ephexin3 expression data, had a significant cumulative risk with respect to patient survival. The 13 survival curves of the transcriptional signatures that correlated with shorter patient survival are shown in Figure 3G. Graphs are ordered according to statistical significance, which was higher for LGG.

### 2.6. Cancer-Specific CMCR-Ephx3-SC Defining Transcriptional Signatures Statistically Linked to Shorter Patient Survival

**Figure 4:**
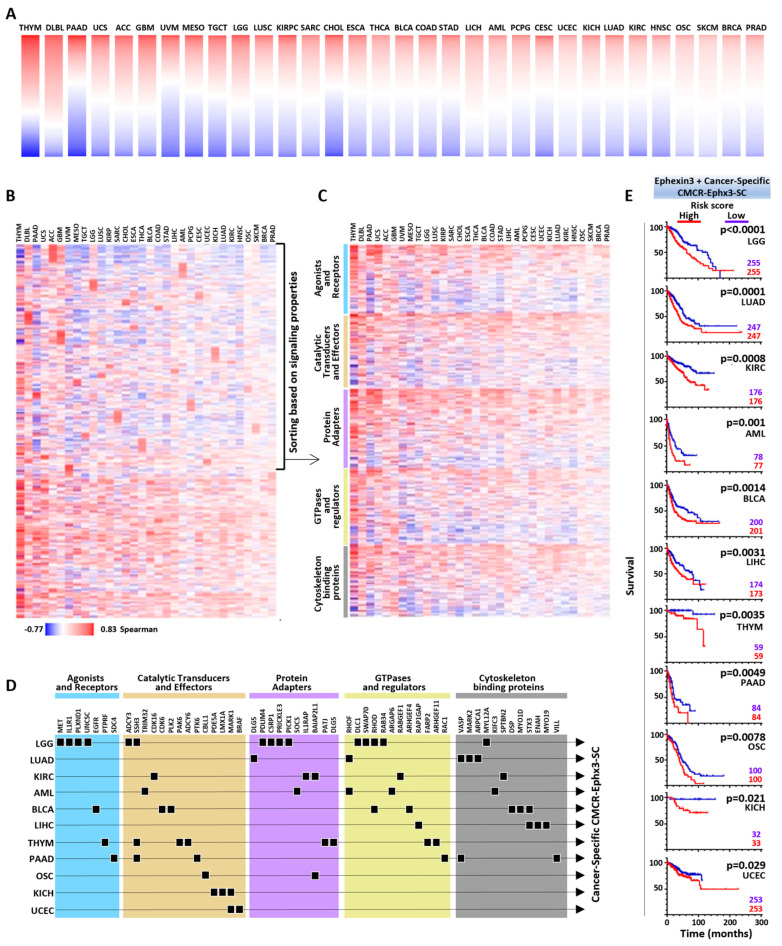
Cancer-specific CMCR-Ephx3-SC transcriptional signatures statistically linked to shorter patient survival. (**A**) Ephx3-SC repertoire in individual heatmaps for each type of cancer. (**B**) Cancer-specific Ephx3-SCs per cancer type. Upper section represents individual signaling companions for Ephexin3. (**C**) Ephx3-SCs organized according to structural and functional criteria. (**D**) Ephx3-SCs correlated with shorter survival in 11 cancer types (arrows) per signaling category. (**E**) Survival curves obtained with cancer-specific Ephx3-SC transcriptional signatures ordered according to their statistical significance. Patients were segregated according to median expression. Multivariate Cox regression analysis from KM plotter.

All cancer types had a group of CMCR-Ephx3-SCs with the highest Spearman correlation values that included some exclusive transcripts not included in the PanCancer list. As a second mining strategy, we focused on the list of cancer-specific CMCR-Ephx3-SCs extracted from the top of transcripts represented by cancer-specific heatmaps (Figure 4A). On average, 188 CMCR-Ephx3-SCs constituted the particular repertoire of each cancer type, all represented together in the heatmap shown in Figure 4B. The upper portion of the heatmap shows highly correlated Ephexin3 signaling partners for each particular cancer type, discernible as mosaic patterns composed of red areas representing cancer-specific CMCR-Ephx3-SC. The lower part of the heatmap depicts CMCR-Ephx3-SCs that, although were identified based on the cancer-specific highest Spearman correlation values (top values of the lists represented as cancer-specific heatmaps in Figure 4A), corresponded to transcripts shared among various cancers. The cancer-specific CMCR-Ephx3-SCs were sorted out based on the signaling properties of the encoded proteins (Figure 4C). Then, we selected the top five of each group and analyzed the Kaplan–Meier survival curves for each one of them. Those with a significant statistical correlation between high expression and shorter patient survival are indicated with square symbols in Figure 4D and the transcriptional signatures for the indicated cancer types are indicated by arrows joining them. Together with Ephexin3, the cancer specific transcriptional signatures were analyzed to detect whether they constitute a higher risk score for each cancer type. Those with statistically significant values are shown as Kaplan–Meier survival curves in Figure 4E. 

### 2.7. CoEssential CMCR-Ephx3-SCs Defining Transcriptional Signatures Statistically Linked to Shorter Patient Survival

**Figure 5:**
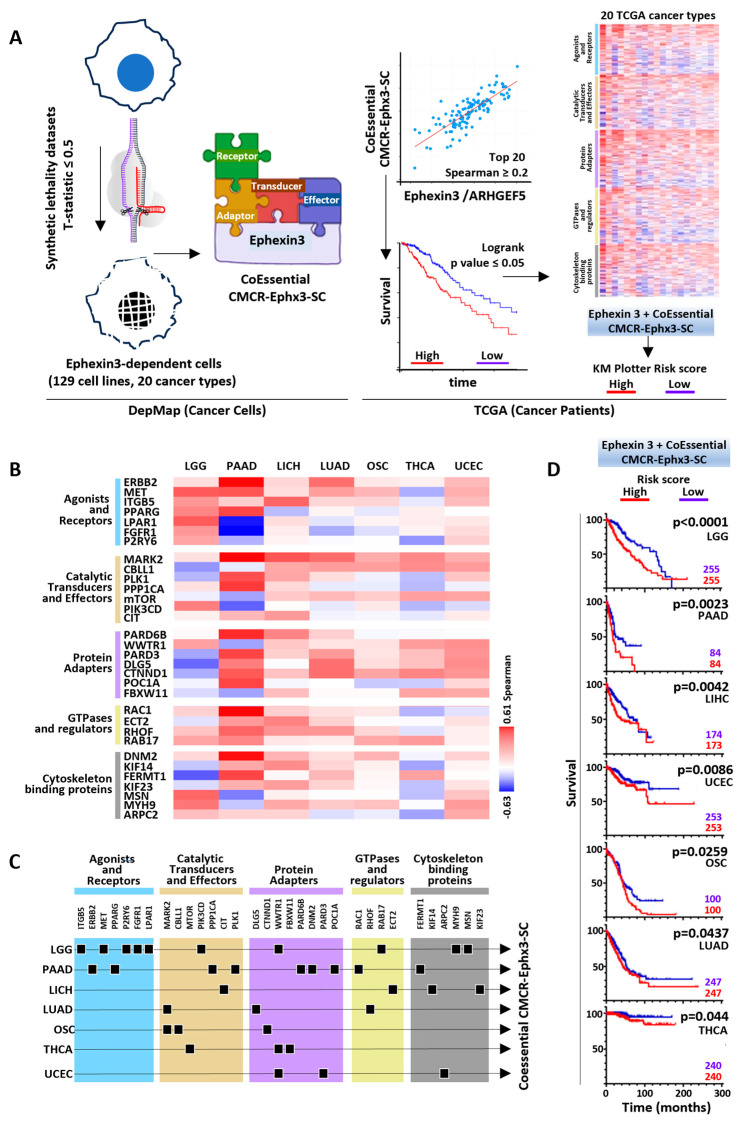
CoEssential CMCR-Ephx3-SC transcriptional signatures linked to shorter patient survival. (**A**) Model of the screening strategy starting from gene dependency in cancer cell lines to correlation with patients’ samples and survival outcome. Right: heatmap of CoEssential CMCR-Ephx3-SCs co-expressed in TCGA studies organized according to structural criteria. (**B**) Selected covulnerabilities that correlate with Ephexin3 in TCGA patients organized according to signaling function. (**C**) Signaling companions that correlate with lower survival in seven cancer types (arrows). Genes were classified according to structural features (color boxes). (**D**) Survival curves of the accumulated risk represented by the co-essential transcriptional signatures. Patients segregated according to median expression. Multivariate Cox regression analyses conducted in KM plotter.

Oncogenic signaling cascades potentially linked to Ephexin3 were investigated by searching for coessential signaling partners in the cell lines sensitive to Ephexin3 knockout (https://depmap.org/portal/gene/ARHGEF5?tab=overview, accessed on 1 November 2021, Figure 2B,C). The systematic knockout of Ephexin3 in hundreds of cell lines, using CRISPR-Cas9 technology, revealed a group of cancer cell lines of various origins in which the lack of Ephexin3 resulted in cell vulnerability. As a third data mining strategy, we investigated the global signaling sensitivities of these 129 cell lines, corresponding to 20 cancer types studied in the TCGA project, in order to identify CoEssential Ephexin3 signaling partners (Figure 5A, left panel). On average, for each cancer type, we identified 468 CoEssential signaling partners. The cancer cell lines in which Ephexin3 was essential corresponded to 20 TCGA cancer types, where we searched for the coexpression of these coessential Ephexin3 signaling partners, selecting those linked to cell migration and cytoskeleton reorganization from the list identified in the DepMap synthetic lethality datasets. Those with higher Spearman correlation values were organized in five groups based on the functionality of the encoded proteins (Figure 5B) and were individually analyzed for their statistical correlation with patient survival. As carried out in the two previous data mining strategies, those with higher expression linked to shorter patient survival were integrated with Ephexin3 in transcriptional signatures (Figure 5C) to analyze their accumulated risk score with respect to patient survival. With this third strategy, we identified seven coessential CMCR-Ephx3-SC transcriptional signatures statistically linked to shorter patient survival (Figure 5D).

### 2.8. CMCR-Ephx3-SC Identified by Independent Data Mining Strategies

**Figure 6:**
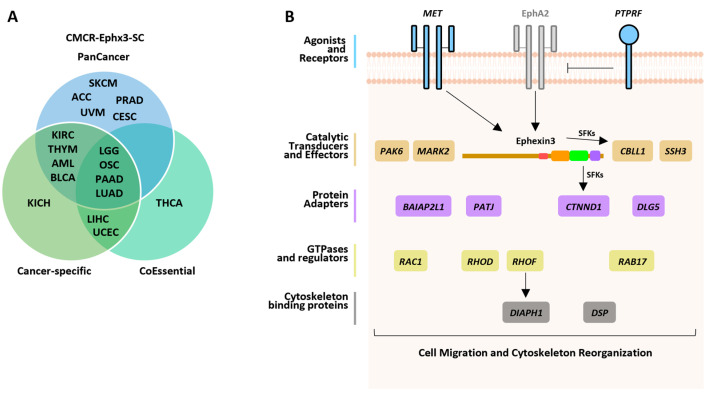
CMCR-Ephx3-SCs in TCGA cancer studies found through different mining strategies and their putative role in signaling cascades integrated by Ephexin3. (**A**) Venn diagram including the TCGA studies with transcriptional signatures that correlated with a higher risk of shorter patient survival identified via the indicated mining strategies. (**B**) Final model depicting a putative signaling cascade integrated by Ephexin3 and CMCR-Ephx3-SC identified as part of transcriptional signatures that correlated with a higher accumulated risk of shorter survival for patients with cancer types identified via at least two out of three mining strategies.

As described, we used three independent systematic approaches to analyze TCGA and DepMap datasets, aiming to identify clinically relevant CMCR-Ephx3-SCs. We identified 31 transcriptional signatures statistically linked to shorter patient survival in 17 cancer types, and 10 of them coincided with at least two data mining strategies (Figure 6A). Various CMCR-Ephx3-SCs were consistently identified in different transcriptional signatures, even corresponding to different cancer types, and these included the tyrosine kinase receptor *MET* and the tyrosine phosphatase receptor *PTPRF*, the serine/threonine kinases *MARK2* and *PAK6*, and the Rho GTPases *RHOD*, *RHOF* and *RAC1* (Figure 6B).

## 3. Discussion

Metastasis, a process critically involving dynamic adjustments of cell shape sustaining the migration of cancer cells and others within the tumor microenvironment, kills most cancer patients [36,37,38]. Oncogenic drivers and microenvironmental cues guide the integration of signaling complexes involving RhoGEFs such as Ephexins, canonical regulators of the actin cytoskeleton, as activators of Rho GTPases that culminate in the assembly of migratory structures [1,2,39]. Given their multidomain structure and catalytic activities, the involvement of Ephexins in clinically relevant oncogenic pathways driving cancer cell dissemination is likely defined by their expression levels and the cancer-specific signaling landscape that accompanies them. To advance our current knowledge on the putative role of Ephexins as oncogenic effectors, we explored their mutational profile and expression in thirty-two cancer types of The Cancer Genome Atlas datasets and their essentiality in hundreds of cell lines of the Cancer Dependency Map datasets. Although different Ephexin genes were altered in various cancer patients, no transcriptional differences were evident that might be attributable to gene amplification or other alterations, indicating that the signaling landscape linked to the oncogenic functions of the different Ephexins seems to be related to their essential signaling partners, particularly those involved in cell migration and cytoskeletal reorganization. 

The positive correlation pattern between Ephexins 1, 2, 3 and 4 suggests some level of redundant or complementary functions, while their negative correlation with respect to Ephexin5 suggests that Ephexin5 functions in particular cells of the tumor microenvironment. We found that Ephexins 1, 2, 3, and 4 correlated with *EPCAM*, a marker of epithelial cells, which, considering the epithelial origin of most cancers, was indicative of the expression of such Ephexins in cancer cells. In contrast, Ephexin5 was mainly expressed in cells of the tumor stroma, including lymphoid, myeloid, and endothelial cells. Consistent with its reported role in developmental angiogenesis, Ephexin5 might participate in oncogenic processes leading to new blood vessel formation [8]. 

We found that only Ephexin3 was essential in multiple cancer cell lines and focused on the characterization of the signaling repertoire of receptors, transducers and effectors, among other signaling partners, highly coexpressed with Ephexin3 in the thirty-two TCGA cancer datasets, pursuing the hypothetical existence of clinically significant transcriptional signatures integrated by Ephexin3 and its signaling partners linked to cell migration. As would be expected for a cancer specific target, the loss of Ephexin3 expression did not generate sensitivity in all cell lines of a particular cancer type, which is consistent with the putative role of Ephexin3 in essential signaling cascades driven by a particular oncogenic landscape. This possibility is consistent with the documented pro-oncogenic mechanisms and signaling complexes involving Ephexin3 [12,14,15,18,40,41,42], which include the integration of signaling complexes composed of growth factor receptors, cytosolic tyrosine kinases, and proteins that interact with the cytoskeleton related to cell proliferation, survival and migration [15,18,41]. This possibility is further consistent with the discovery of Ephexin3 as an active oncogene encoded by a truncated clone lacking the amino-terminal region [17,43]. Although speculative, Ephexin3 functional singularity might be attributable to its main structural differences with respect to its closest homologues, which mainly reside in the amino-terminal region of Ephexin3, a region that within the group of Ephexins is considered a regulatory node [44]. Although it might seem counterintuitive that signaling proteins driving cytoskeletal reorganization and cell migration play a role in cancer cell proliferation and survival, various RhoGEFs and Rho GTPases are known to activate transcriptional programs driving these processes [45,46,47,48]. In addition, actin polymerization in cancer cells results in a decreased inhibition of the transcription factor MRTF-A, which is sequestered by unpolymerized actin [49]. Upon actin polymerization, MRTF-A forms a complex with SRF, activating a transcriptional program that promotes mesenchymal transition [50], resulting in reduced apoptosis, and increased cell survival, tumorigenesis and drug resistance [50]. Moreover, actin polymerization controls gene expression via the Hippo pathway, critically involved in mesenchymal transition and the survival of cancer cells [51,52,53]. In the case of Ephexin3, He and colleagues demonstrated that this RhoGEF (ARHGEF5) is critically required for lung cancer cell adhesion and migration, but also proliferation and invasion [41]. At the molecular level, ARHGEF5 expression was linked to cyclin D and MMP2 expression, as shown in knockdown cells which produced smaller and less metastatic tumors in mice, whereas the opposite effects were found in cells overexpressing ARHGEF5 [41]. In colorectal cancer cells, Ephexin3/ARHGEF5 was required for their invasive and in vivo metastatic activity [15]. The transcriptional effects of Ephexin3/ARHGEF5 seem to be cell type specific, as in endothelial cells this RhoGEF contributes to MRTF-A expression in a process driving mesenchymal transition in response to TGFβ [54], whereas the transforming version of this RhoGEF, known as TIM, stimulates SRF and AP-1 transcriptional activity [43]. 

Regarding the clinically significant signaling cascades integrated by Ephexin3, the cumulative risk analysis of the transcriptional signatures revealed their potential importance in various cancer types. We identified a repertoire of receptors, transducers and effectors that lead cytoskeleton remodeling and cancer cell migration, all of them statistically linked to shorter patient survival. The coessential signatures are of particular interest as they come from covulnerabilities detected in cancer cell lines that corresponded to signaling proteins that were coexpressed with Ephexin3 in cancer patients and were statistically linked to shorter patient survival. This would indicate that these signaling cascades, in addition to being essential, might be related to tumor progression. The strategy that led us to identify Ephexin3-linked transcriptional signatures, based on the relational data mining of mechanistically linked signaling elements, sets the basis to postulate them as potential oncogenic networks. However, it is important to keep in mind that their putative role in Ephexin3 signaling and their contribution to cancer progression has to be functionally tested before reaching mechanistic conclusions. Given that some of these signaling molecules are essential in cancer cells corresponding to aggressive tumors for which clinically useful treatments are very limited, such as pancreatic [55,56] and lung cancers [57], their identification raises interesting possibilities to investigate their potential as biomarkers and pharmacological targets. The transcriptional signatures, analyzed via Cox regression as elements that together contribute to an accumulated risk of shorter patient survival, only included transcripts that individually, as determined via conventional Kaplan–Meier analysis, were statistically linked to shorter patient survival. The rational was that, together, these elements of the Ephexin3 signaling landscape represent a potential collective contribution to the mechanistic basis of cancer cell dissemination. In general terms, given that more precise diagnosis can be reached when multiple diagnostic elements are considered, we translated this concept, looking for integrated signaling networks putatively linked to the underlying processes by which cytoskeletal reorganization and cell migration contribute to cancer progression. Among the Ephexin3 signaling partners identified in various cancer types, the receptor tyrosine kinase Met stands out, as it has been fully documented as a participant of metastatic cancer [58,59,60]. Other Ephexin3 signaling companions include molecular switches directly involved in the reorganization of the actin cytoskeleton such as RhoD [61,62] and RhoF [63] and effectors of Rho GTPases such as PAK6 [64,65] and DIAPH1 [66,67], further supporting the putative role of Ephexin3 as a signaling platform. In conclusion, the identification of clinically relevant signaling signatures found via independent rational data mining strategies, focusing on signaling proteins involved in cytoskeleton reorganization and cell migration, raises hypothesis-driven questions that warrant future investigations oriented toward addressing their participation in oncogenic processes in the context of the putative role of Ephexin3 as an effector of metastatic signaling cascades.

## 4. Materials and Methods

### 4.1. Ephexins in TCGA Studies 

The mRNA expression (RSEM, (HiSeq_RNAs Ilumina, normalized)) and copy number variation of Ephexins (1–5) were analyzed in the cBioPortal platform (https://www.cbioportal.org/, accessed on 1 November 2021); these Ephexins were Ephexin1 (*NGEF*), Ephexin2 (*ARHGEF19*), Ephexin3 (*ARHGEF5*), Ephexin4 (*ARHGEF16*) and Ephexin5 (*ARHEF15*) for the 32 cancer studies of the TCGA. Alterations included “Diploid”, “Gain” “Shallow Deletion” “Deep Deletion” and “Amplification”.

### 4.2. Signaling Transcripts Coexpressed with Ephexins in TCGA Studies

For each Ephexin and cancer type, a coexpression gene list was downloaded and labeled with the corresponding codifying protein information that served to filter those participating in signaling cascades. The correlation for a given Ephexin was compared with that for the rest of the Ephexins per cancer study. 

For the Ephexin3 signaling companions, the top 10% of the correlated signaling transcripts were selected. In cancer types in which 10% of the extracted transcripts contained Spearman’s correlation values of less than 0.2, these were eliminated. We filtered those coexpressed transcripts that code for proteins participating in the reorganization of the cytoskeleton and potentially cell migration. Gene Onthology functional tags related to cell migration and the formation of specialized structures were searched in the Panther platform (https://pantherdb.org/about.jsp, accessed on 26 July 2022) and then in the Metascape platform (https://metascape.org/gp/index.html#/main/step1, accessed on 11 October 2022) to identify enriched GO terms. Signaling companions were grouped into five groups according to a functional classification: ligands and receptors, proteins with catalytic activity, adapter proteins, proteins involved in the GTPase cycle and proteins that interact with the cytoskeleton. Signaling companions were organized in descending order of cumulative Spearman’s values. Columns were rearranged in descending order according to Spearman’s cumulative value from left to right. Coexpression values were shown in heatmaps generated with the Clustergrammer package developed by Ma’yan Lab. “Jupiter Lab” was downloaded from Anaconda (https://www.anaconda.com/, accessed on 8 March 2022).

For the PanCancer CMCR-Ephx3-SC transcriptional signatures, a signaling core was integrated with the Signaling companions of the 32 cancer studies. For the Cancer-Specific CMCR-Ephx3-SC transcriptional signatures, the signaling companions of each cancer study were analyzed.

### 4.3. Ephexins’ Correlation with Cell Type Markers 

Coexpression lists were downloaded per cell type marker: *EPCAM* for epithelial cells, *PECAM* for endothelial cells, *PTPRC* for lymphoid cells, *ITGAM* for myeloid cells and *FAP* for fibroblasts. Ephexins’ correlation with each marker was collected and illustrated in heatmaps for the 32 cancer types.

### 4.4. Ephexins’ Essentiality and CoEssential Partners

The Ephexins’ CRISPR effect (Public 21Q4 + Score, Chronos) was evaluated in cancer cell lines of different tissues on the DepMap database platform (https://depmap.org/portal/, accessed on 1 November 2021). T statistic values were ordered from lowest to highest to select those equal or less than −0.5. Covulnerabilities to Ephexin3 loss were downloaded per cell line and labeled with the information of the corresponding protein to select those participating in signaling cascades. CoEssential genes were harmonized with coexpression data of patients from the TCGA project and organized according to structural and functional characteristics for the CoEssential CMCR-Ephx3-SC signature.

### 4.5. Ephexin3 Transcriptional Signatures

Transcriptional signatures were formed by Ephexin3 and signaling companions (Ephx3-SC) whose individual expression was statistically correlated with shorter patient survival. For individual survival curves, patients were segregated according to “Auto select best cutoff” and analyzed via Cox regression. This procedure was applied for each type of cancer and the identified signaling partners using any of the three mining strategies: PanCancer, Cancer-Specific or CoEssential. The accumulated risk of the transcriptional signatures (Ephexin3 and companion genes altogether) was evaluated in KM plotter (https://kmplot.com/analysis/, accessed on 30 November 2022) in the “Custom” section, with a multivariate Cox regression analysis, dividing patients by the median expression of the collective genes per cancer study.

### 4.6. Statistical Analysis 

Spearman’s correlation coefficient values were calculated on the cBioportal platform. Individual and multivariate Cox regression analyses were conducted in KM plotter and verified in GraphPad Prism (6.01).

## Data Availability

All patient data included in the manuscript were obtained from the TCGA Research Network: https://www.cancer.gov/tcga, accessed on 1 November 2021. Synthetic lethality data were obtained from the DepMap public datasets, https://depmap.org/portal/home/#/, accessed on 1 November 2021.
